# Risk Assessment of Chemical Mixtures in Foods: A Comprehensive Methodological and Regulatory Review

**DOI:** 10.3390/foods15020244

**Published:** 2026-01-09

**Authors:** Rosana González Combarros, Mariano González-García, Gerardo David Blanco-Díaz, Kharla Segovia Bravo, José Luis Reino Moya, José Ignacio López-Sánchez

**Affiliations:** Escuela Superior de Ingeniería y Tecnología (ESIT), Universidad Internacional de La Rioja-UNIR, Avenida de la Paz, 137, 26006 Logroño, Spain; rosana.gonzalez@unir.net (R.G.C.); mariano.gonzalezgarcia@unir.net (M.G.-G.); gerardo.blanco@unir.net (G.D.B.-D.); kharla.segoviab@unir.net (K.S.B.); joseluis.reino@unir.net (J.L.R.M.)

**Keywords:** combined exposure, mixture risk assessment, hazard index (HI), relative potency factor (RPF), toxic equivalents (TEQ), Maximum Cumulative Ratio (MCR), margin of exposure (MOE), combined MOE (MOET), EFSA, EPA, WHO/FAO, Total Diet Studies (TDS), human biomonitoring (HBM), uncertainty communication

## Abstract

Over the last 15 years, mixture risk assessment for food xenobiotics has evolved from conceptual discussions and simple screening tools, such as the Hazard Index (HI), towards operational, component-based and probabilistic frameworks embedded in major food-safety institutions. This review synthesizes methodological and regulatory advances in cumulative risk assessment for dietary “cocktails” of pesticides, contaminants and other xenobiotics, with a specific focus on food-relevant exposure scenarios. At the toxicological level, the field is now anchored in concentration/dose addition as the default model for similarly acting chemicals, supported by extensive experimental evidence that most environmental mixtures behave approximately dose-additively at low effect levels. Building on this paradigm, a portfolio of quantitative metrics has been developed to operationalize component-based mixture assessment: HI as a conservative screening anchor; Relative Potency Factors (RPF) and Toxic Equivalents (TEQ) to express doses within cumulative assessment groups; the Maximum Cumulative Ratio (MCR) to diagnose whether risk is dominated by one or several components; and the combined Margin of Exposure (MOET) as a point-of-departure-based integrator that avoids compounding uncertainty factors. Regulatory frameworks developed by EFSA, the U.S. EPA and FAO/WHO converge on tiered assessment schemes, biologically informed grouping of chemicals and dose addition as the default model for similarly acting substances, while differing in scope, data infrastructure and legal embedding. Implementation in food safety critically depends on robust exposure data streams. Total Diet Studies provide population-level, “as eaten” exposure estimates through harmonized food-list construction, home-style preparation and composite sampling, and are increasingly combined with conventional monitoring. In parallel, human biomonitoring quantifies internal exposure to diet-related xenobiotics such as PFAS, phthalates, bisphenols and mycotoxins, embedding mixture assessment within a dietary-exposome perspective. Across these developments, structured uncertainty analysis and decision-oriented communication have become indispensable. By integrating advances in toxicology, exposure science and regulatory practice, this review outlines a coherent, tiered and uncertainty-aware framework for assessing real-world dietary mixtures of xenobiotics, and identifies priorities for future work, including mechanistically and data-driven grouping strategies, expanded use of physiologically based pharmacokinetic modelling and refined mixture-sensitive indicators to support public-health decision-making.

## 1. Introduction

Over the last 15 years, mixture risk assessment for food xenobiotics has progressed from simple screening approaches towards component-based and probabilistic methodologies. This evolution reflects the recognition that consumers experience concurrent, lifelong dietary exposures to multiple chemicals—or “cocktails”—that may yield effects not captured by single-substance assessments [1,2,3,4,5]. Globalisation of food chains and use of pesticides, food contact materials and industrial chemicals have made anthropogenic substances ubiquitous in environmental and food matrices, with diet a dominant exposure route for pesticides, process contaminants, mycotoxins, metals and per- and polyfluoroalkyl substances (PFAS) [3,4,6,7,8,9]. Within this broader exposome, the “combined exposure” paradigm explicitly addresses real-world mixtures and the “cocktail effect”, under which chemicals can interact additively, synergistically or antagonistically [1,5,10,11,12,13,14].

At the toxicological level, mixture behavior is framed by two reference models: concentration (dose) addition and independent action (response addition) [5,12]. Dose addition posits that components act as dilutions of one another, sharing a similar mode of action, whereas independent action assumes statistically independent effects for chemicals acting through different pathways. A substantial experimental corpus indicates that, at low effect levels, predictions from these models often converge, with many environmental mixtures conforming reasonably well to dose addition even when modes of action are not strictly identical [5,12,15,16]. True synergy and strong antagonism do occur, but systematic evaluations indicate they are quantitatively limited and relatively infrequent at environmentally realistic doses, particularly for pesticide mixtures [17,18]. These findings underpin the widespread use of dose-additive, component-based methods as a conservative default for cumulative dietary risk assessment, while recognizing that interaction “hotspots” and data gaps remain [19,20,21].

Over the same period, major agencies have moved from largely theoretical discussions of chemical mixtures to operational cumulative risk assessment (CRA) frameworks. The European Food Safety Authority (EFSA), the U.S. Environmental Protection Agency (EPA) and joint FAO/WHO initiatives now share a backbone based on tiered assessment, biologically informed grouping and default dose addition for substances with similar or overlapping effects [1,2,7,22,23,24,25,26]. EFSA has pioneered developments in the food domain, establishing cumulative assessment groups (CAGs) for pesticides targeting the nervous system, thyroid and craniofacial development and embedding CRA in routine opinions [27,28,29,30,31]. EPA’s common mechanism groups and relative potency factor (RPF) methodology for organophosphate and other pesticides represent a parallel, strongly quantitative tradition [25,26,32]. FAO&WHO, through its Framework and Guidance for combined exposure to multiple chemicals in food, has sought to harmonize principles and provide scalable tools for countries with differing data infrastructures [7].

Thus, combined risk assessment for food-related chemical mixtures can be structured around two complementary approaches: the whole-mixture approach and the component-based approach. In the whole-mixture approach, a real mixture (e.g., a formulation or complex environmental extract) is treated as the test item, and hazard and risk are characterized directly from the observed effects of the entire mixture. By contrast, the component-based approach requires detailed information on each constituent’s identity, concentration, and toxicity, including its assumed or established mode of action (MoA). For component-based assessments, chemicals that share similar MoAs and act on the same toxicological target are typically evaluated using dose-addition models, often combined with toxicity or potency factors and grouping into assessment groups. Chemicals with dissimilar MoAs that nonetheless contribute to a common endpoint are handled under independent-action concepts, which can be implemented either as response addition (probabilistic aggregation of component risks) or effect addition (summation of measured biological responses, with adversity boundaries commonly defined by NOAEL-type reference points). In practice, key elements of combined-risk characterization therefore include: analysis of co-exposure and co-occurrence patterns, grouping of substances into assessment groups, application of dose-addition models with toxicity/potency factors, and use of response- or effect-addition schemes depending on MoA similarity. Tralau et al. also emphasise the generation of co-exposure patterns for specific consumer subgroups (e.g., defined by lifestyle or dietary habits) to prioritize mixtures for assessment, while highlighting persistent data gaps and uncertainties in human relevance that complicate component-based evaluations. Real-world concerns such as reported cumulative hepatotoxicity from pesticide combinations are used to justify the need for proactive, methods-based mixture testing and exposome-informed co-exposure analyses to identify mixtures where hazards may otherwise be overlooked [33].

In food safety, implementation of these frameworks depends critically on robust exposure data streams. Total Diet Studies (TDS) are central for characterizing chronic dietary exposure “as eaten” by selecting foods that cover ≥85–90% of intake, preparing them as consumed and pooling them into composite samples for multi-analyte analysis [34,35]. Harmonized guidance from WHO, FAO and EFSA, refined in European projects such as TDS-Exposure, has consolidated best practice on sampling, home-style preparation, pooling and left-censor handling [36,37,38]. National TDS implementations show that such designs can yield realistic, policy-relevant exposure estimates for a broad spectrum of contaminants while maintaining analytical feasibility [39,40,41,42,43]. At the same time, loss of product-level resolution in TDS motivates their integration with conventional monitoring and model-based tools in cumulative assessments [44,45,46].

Complementing external exposure data, human biomonitoring (HBM) quantifies internal exposure to diet-related xenobiotics in biological matrices, integrating all routes and sources of uptake [6,47,48]. Large initiatives such as HBM4EU and WHO/Europe have harmonized protocols, quality assurance and health-based guidance values (HBM-GVs), enabling mixture-aware assessments based on internal concentrations rather than external intake alone [49,50,51,52,53]. Applications to PFAS, phthalates, bisphenols and mycotoxins illustrate how HBM links exposure, internal dose and risk characterization, combining serum or urinary measurements with PBPK modelling, guidance values and Total Diet Study data [8,54,55,56,57,58,59,60,61,62,63,64,65,66].

On this foundation, a suite of quantitative metrics has been operationalized to assess mixture risk under default dose additivity. These range from screening tools like the Hazard Index (HI) to refined approaches such as Relative Potency Factors (RPF), Toxic Equivalents (TEQ), the Maximum Cumulative Ratio (MCR), and the combined Margin of Exposure (MOET) [20,21,30,32,54,55,67,68,69,70,71,72,73,74,75,76,77,78,79,80,81,82,83,84].

Despite these advances, mixture risk assessment remains characterized by substantial uncertainty. Parameter uncertainty arises from incomplete or variable toxicity data, co-exposure correlations and toxicokinetic variability; model uncertainty from metric choice, additivity assumptions and extrapolation frameworks; and scenario uncertainty from decisions on problem formulation, grouping, populations and exposure windows [20,21,85,86,87]. Recent guidance from EFSA and the U.S. National Academies stresses that explicit, tier-appropriate uncertainty analysis is integral to mixture assessment and that local and global sensitivity analyses are essential for identifying dominant “risk drivers” [85,86,87,88]. Concurrently, research on risk communication highlights that reporting binary outcomes (e.g., HI < 1) is insufficient; stakeholders require transparent information on variability, residual uncertainty and the conditional nature of model assumptions, conveyed through clear narratives, ranges and graphical tools [89,90,91,92]. Thus, a consolidated framework integrating toxicology, exposure science, and regulatory policy is currently lacking.

Within this evolving landscape, this review synthesizes methodological and regulatory developments in mixture risk assessment for food xenobiotics. Specifically, it: (i) critically examines core combined-risk metrics and their underlying assumptions; (ii) compares leading regulatory frameworks for cumulative assessment (EFSA, EPA, WHO/FAO) and their degree of convergence; (iii) summarizes best practices and challenges for integrating Total Diet Studies and Human Biomonitoring into mixture assessments; (iv) discusses how “cocktail effect” theory is translated into operational grouping strategies, metrics and decision rules; and (v) outlines an agenda for uncertainty analysis, communication and future research, including the role of PBPK modelling and data-driven grouping approaches [1,14,19,23,30,50,53,87,93]. By focusing specifically on food-relevant mixtures and integrating exposure, toxicology and regulatory perspectives, the review aims to support a transition from single-chemical paradigms to transparent, tiered and uncertainty-aware frameworks capable of managing real-world dietary “cocktails” of xenobiotics in a proportionate and scientifically robust manner.

Although several recent publications have reviewed specific aspects of chemical mixture risk assessment, most have tended to concentrate on either methodological innovations, regulatory developments, or selected exposure domains in isolation. The present review aims to provide a clearly differentiated study and added value by offering a genuinely integrative perspective. It connects core methodological foundations (including metrics, grouping strategies and toxicological assumptions) with the implementation approaches adopted by major international regulatory bodies (EFSA, U.S. EPA and FAO/WHO), and explicitly links these frameworks to the practical evidence base afforded by Total Diet Studies and Human Biomonitoring. Moreover, this review goes beyond descriptive synthesis by discussing best practices, common pitfalls, uncertainty management and risk communication, and by providing structured guidance on the applicability and prioritization of different cumulative assessment methodologies. This practice-oriented and analytically grounded perspective is intended to support both researchers and risk assessors in the effective and informed implementation of mixture risk assessment in food-related contexts.

The review is structured as follows. Section 2 describes the structured methodology used to identify and select guidance documents, methodological contributions and case studies. Section 3 introduces the core methodological foundations for mixture risk assessment, including key combined-risk metrics, toxicological models and grouping concepts. Section 4 compares major regulatory frameworks developed by EFSA, the U.S. EPA and FAO/WHO. Section 5 summarizes how Total Diet Studies and Human Biomonitoring provide exposure data streams to support mixture risk assessment. Section 6 provides a structured Discussion, synthesizing best practices, sources and management of uncertainty, approaches for effective risk communication, and forward-looking priorities for the further development and implementation of mixture risk assessment. Finally, Section 7 provides conclusions and an outlook on future research and regulatory priorities.

## 2. Methodology

This review follows a structured and transparent approach to identifying, selecting, and synthesizing the literature on risk assessment of chemical mixtures in foods over the period 2010–2025. The focus is on methodological and regulatory developments, as well as on capturing representative case studies. The primary aim was to map concepts, frameworks and metrics across regulatory and scientific domains.

### 2.1. Identification of Key Guidance and Framework Documents

Before performing topic-specific database searches, a priori list of key regulatory and scientific framework documents on chemical mixture risk assessment was compiled. These included EFSA guidance and applications, combined exposure, cumulative assessment groups, and uncertainty analysis [27,28,29,82,88], FAO/WHO framework and guidance documents on cumulative risk assessment of chemicals in food [23], broader methodological overviews [1,80,81,91,92,93], U.S. EPA guidance on cumulative and combined exposures [24,25,26], and global policy reports on chemicals management [7]. Forthcoming EFSA perspectives on future directions in chemical mixture risk assessment were also considered [31]. These documents were used as seed references to define the conceptual scope of the review and to identify additional primary studies and applied case examples through backward and forward citation tracking.

### 2.2. Bibliographic Search Strategy and Information Sources

All contributing authors conducted coordinated bibliographic searches, with Web of Science (Core Collection) as the common primary database. Depending on the thematic section, searches were complemented with the EFSA Journal platform and the WHO Institutional Repository for Information Sharing (IRIS). Across topics, the searches targeted peer-reviewed literature and authoritative reports from international and national agencies (e.g., EFSA, FAO/WHO, U.S. EPA, WHO, HBM4EU, UNEP).

Search strategies were tailored to each component of the review:

For regulatory frameworks and methodological guidance, combinations of terms such as “chemical mixtures”, “risk assessment”, “combined exposure”, “cumulative exposure”, “EFSA guidance”, “cumulative assessment group”, “hazard index”, “relative potency factor (RPF)”, “point of departure (POD)”, “PBPK models” and “mixture toxicity” were used in Web of Science and Scopus, typically yielding between ~200 and 500 records per search topic before screening.

For Total Diet Studies (TDS), a targeted search in Web of Science was performed using the terms “Total Diet Studies”, “TDS”, “evolution”, “methodology” and “results”. This search initially retrieved 33 records; after screening and removal of non-relevant or redundant items, 12 references were retained for the TDS section. For the “cocktail effect” and mixture-toxicity concepts, searches were conducted using specific terms “risk assessment of chemical mixtures”, “HI”, “MOET”, “CAG”, “POD” and “cocktail effect”.

For human biomonitoring (HBM) and dietary origin, the string used was: “Human biomonitoring” AND (“dietary exposure” OR “dietary origin” OR “total diet study” OR “food safety”) AND (“mixture risk” OR “HI” OR “MOET” OR “combined exposure”) AND (“HBM4EU” OR “PFAS” OR “phthalates” OR “bisphenols” OR “mycotoxins” OR “PBPK modeling” OR “biomarkers”).

Across all topics, additional references were identified by “snowballing” (screening the reference lists of key papers and guidance documents and identifying citing articles) to capture relevant methodological or regulatory contributions not retrieved in the initial keyword searches.

### 2.3. Eligibility Criteria and Study Selection

Inclusion and exclusion criteria were harmonized while allowing topic-specific refinement. Thus, inclusion criteria (general): (i) Peer-reviewed articles with a DOI or official technical reports/guidance from recognized bodies (EFSA, FAO/WHO, U.S. EPA, WHO, UNEP, HBM4EU); (ii) Primary focus on chemical mixtures, combined or cumulative exposure, or mixture toxicity, with clear relevance to food, dietary exposure, human health or, when informative for methodological aspects, environmental health; (iii) Publications mainly between 2010 and 2025, consistent with the nominal timeframe of the review, with targeted inclusion of key pre-2010 documents when essential to describe the evolution of frameworks and concepts.

Exclusion criteria: (i) Studies focused exclusively on the toxicity of single substances without a mixture, cumulative or combined-exposure component; (ii) Articles without accessible full text (open-source or not), conference abstracts, preprints and non-peer-reviewed opinion pieces without institutional backing; (iii) Studies limited to purely analytical method development or occurrence data without any link to human exposure or risk assessment; (iv) Duplicated records (within and across databases and authors’ searches) and papers judged not relevant to the methodological or regulatory questions addressed in this review.

Title and abstract screening were performed, followed by full-text assessment for uncertain cases. Overlaps between topic-specific reference lists were then resolved by cross-checking and eliminating duplicates. After iterative screening, snowballing and deduplication, a final set of 124 unique references was retained for detailed analysis in the present review.

For each included reference, information pertinent to the aims of the review was extracted: (i) type of mixture and exposure with emphasis on foods and dietary pathways; (ii) risk-assessment framework and metric employed (e.g., HI, RPF, POD, MOET, CAGs, PBPK modeling); (iii) regulatory or institutional context (e.g., EFSA, FAO/WHO, U.S. EPA applications), and (iv) key methodological contributions, limitations and implications for future practice. Extracted information was synthesized qualitatively within thematic sections.

AI-based tools were used in a supportive, non-decisive manner and did not replace expert judgement at any stage. The selection, critical appraisal, interpretation, and final drafting were exclusively performed by the authors based on the primary literature and official reports.

## 3. Methodological Foundations for Mixture Risk Assessment in Foods

### 3.1. Key Metrics for Combined Risk Assessment

**Figure 1:**
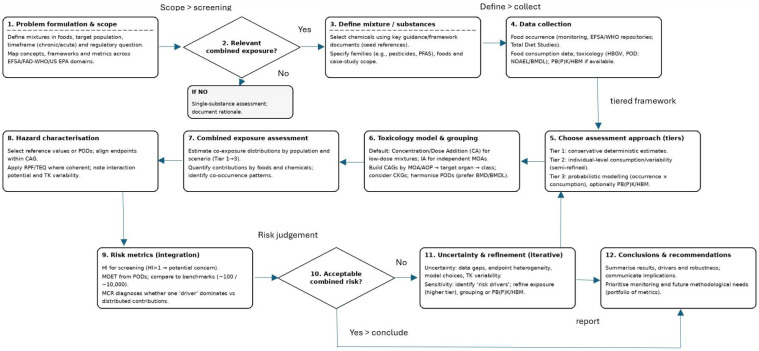
Schematic overview summarizing the stepwise workflow of chemical mixture risk assessment in foods.

Assessing combined chemical risk presents a major challenge for toxicology, given that consumers are chronically exposed to complex mixtures rather than isolated substances [30,94]. Traditional single-substance assessments therefore underestimate risk aggregation and cumulative effects. In response, a set of component-based metrics has been developed to operationalize mixture toxicity under the overarching assumption of dose (concentration) additivity and, where possible, common modes or targets of action. Prominent tools include the Hazard Index (HI), Relative Potency Factors (RPF) and Toxic Equivalents (TEQ), and combined exposure indicators such as the Maximum Cumulative Ratio (MCR) and Margin of Exposure for Total (MOET) [20,21,54,55,67,95]. Their appropriate use is now central to EFSA, US EPA and other agencies’ tiered frameworks for cumulative risk assessment (see Figure 1) [78,82,96].

**Figure 2:**
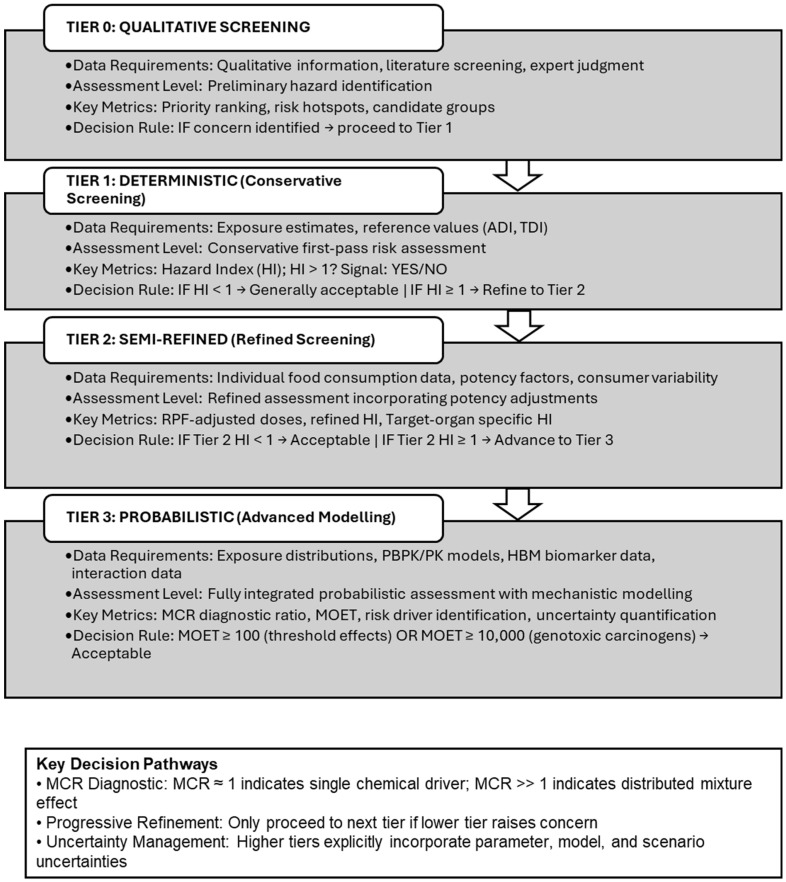
Tiered Assessment Framework for Cumulative Risk Assessment of Food Xenobiotics.

At the exposure level, EFSA (2013) [22] proposed a three-tier paradigm (Figure 2) ranging from conservative deterministic estimates (Tier 1), through semi-refined calculations using individual-level consumption data and variability across consumers (Tier 2), to fully probabilistic assessments (Tier 3) that combine distributions of chemical occurrence with food-consumption patterns. Higher-tier assessments may explicitly integrate spatial–temporal variability, physiologically based toxicokinetic models that link external exposure to internal dose, and, where available, human biomonitoring data and biomonitoring equivalents to characterize internal exposure distributions for mixture risk assessment.

The Hazard Index (HI) is the most established screening tool for mixture risk in food and environmental matrices. It is based on the sum of individual Hazard Quotients (HQ_i_), each defined as the ratio of an exposure estimate to a health-based reference value (e.g., ADI, TDI or reference dose):

$$HI=\sum HQi=\sum (\frac{{Exposure}_{i}}{{Reference\;value}_{i}})$$

Under dose-addition, a HI > 1 is typically interpreted as a signal of potential concern that warrants refined assessment or risk-management scrutiny [76,95,96]. Because it only requires exposure distributions and reference values, HI is particularly attractive for low-tier assessments and for heterogeneous datasets where detailed toxicological information or interaction data are sparse [20,21,67]. Its transparency and conservative nature explain its wide uptake in regulatory guidance from EFSA and US EPA for cumulative exposure scenarios [54,55,97].

However, the apparent simplicity of the HI masks several important limitations. First, reference values are derived using different datasets, endpoints, uncertainty factors and protective assumptions, so summing HQs can obscure heterogeneity in underlying data quality and embedded safety margins [20,21]. Second, HI is a relative indicator of proximity to reference values rather than a predictor of specific biological responses, and it does not explicitly incorporate synergistic, antagonistic or other interaction terms [80,81,98]. Third, the requirement for common or at least compatible endpoints may lead to focusing on a limited set of critical effects while overlooking broader systemic outcomes [76]. Recent developments aim to increase the mechanistic fidelity of HI-type approaches by integrating toxicokinetic information and physiologically based (pharmaco)kinetic (PB(P)K) models, thereby refining internal dose metrics and reducing reliance on external exposure surrogates [67,98,99]. Even with these constraints, HI remains a pivotal entry-level instrument for benchmarking concurrent exposures in food and environmental risk assessment, especially when mixture toxicity is considered alongside other evidence streams [30,96].

EFSA’s 2013 analysis explicitly formalized the additivity assumptions that underpin most combined-risk metrics. For chemicals sharing a common target or mode of action, dose addition is applied, whereas response addition is reserved for components acting through independent modes of action [22]. Thus, although easy to apply and anchored in health-based guidance values, the Hazard Index is only a relative indicator of joint risk: it does not predict the magnitude of health effects when group HBGVs are exceeded and is restricted to chemicals for which such guidance values are available. To address end-point specificity, the Target-organ Toxicity Dose concept refines the HI by using endpoint-specific reference points, leading to separate HIs for different organs or critical effects. Where robust interaction data exist, EFSA also described extensions such as interaction-based HIs and binary-interaction modifiers, which adjust individual hazard quotients to reflect toxicokinetic or toxicodynamic departures from simple additivity [22].

Relative Potency Factors (RPF) and Toxic Equivalents (TEQ) provide more refined tools where mixtures share a common mode of action and robust dose–response data are available. In the RPF framework, each component is normalized to an index compound based on a shared endpoint and comparable study designs; exposures are then expressed as “index-compound equivalents” that can be summed to obtain a total equivalent dose [21,25,26,32]. The TEQ concept is a specialized implementation of this approach and underpins the Toxic Equivalency Factor (TEF) system used for dioxins and dioxin-like PCBs, which act through activation of the aryl hydrocarbon receptor (AhR) [68,71]. Analogous RPF/TEQ-type schemes have been explored for other chemical families such as PFAS, organophosphate insecticides and further emerging contaminants with similar toxicodynamic profiles [21,69,70,71,80,81].

The robustness of RPF and TEQ metrics depends critically on the choice of index compound, the quality and coherence of dose–response data across mixture constituents, and the validity of key assumptions [21,25,26,32]. In particular, dose additivity is typically assumed, so non-additive interactions may be missed [69,70]. Moreover, toxicokinetic and inter-individual variability can undermine the comparability of external doses, especially when extrapolating across life stages or populations. To address these challenges, recent work has integrated PBK models and toxicokinetic data into RPF/TEQ derivations, improving internal-dose equivalence across chemicals [68,69,70]. In parallel, computational toxicology resources such as the US EPA’s CompTox Chemicals Dashboard, combined with in vitro assays, QSAR models and read-across approaches, are increasingly used to support or approximate potency factors for data-poor chemicals [72,95]. These advances extend the reach of RPF/TEQ approaches beyond classical dioxin-like compounds towards broader groups of emerging pollutants, while retaining regulatory compatibility [54,55,71].

Beyond these component-scaled metrics, combined exposure indicators such as the Maximum Cumulative Ratio (MCR) and Margin of Exposure for Total (MOET) offer complementary perspectives on mixture risk. MCR is defined as the ratio between the combined risk estimate for the mixture and the risk estimate for the single most contributing component. Values close to 1 indicate that overall risk is dominated by one “driver” compound and that a single-substance approach may capture most of the mixture hazard, whereas higher MCR values signal more distributed contributions and highlight the added value of full mixture modelling [21,80,81,84]. In practice, MCR is mainly used as a diagnostic metric to prioritise which mixtures or scenarios require more advanced assessment, including in food, environmental and other high-risk process-industry contexts [30,84].

The MOET consolidates individual Margins of Exposure (MOE_i_) for mixture components without simply multiplying or compounding generic assessment factors. It is typically defined as:

$$\frac{1}{MOET}=\sum (\frac{1}{{MOE}_{i}})$$

where MOE_i_ is the ratio between a toxicological point of departure (POD_i_) and exposure_i_ for each component [75,80,81]. Interpretation usually follows pragmatic benchmarks aligned with regulatory precedent: for example, MOET ≥ 100 is often considered adequate for threshold toxicants, whereas MOET ≥ 10,000 is a common target for genotoxic carcinogens, mirroring single-substance risk assessment practices [73,77,91,92]. A key strength of MOET is that it aggregates margins in a way that respects their probabilistic meaning and avoids double-counting uncertainty factors across components [21,75]. However, its implementation requires comparable and reasonably harmonized PODs across substances—ideally benchmark dose (BMD)-based—which can be a substantial constraint when toxicological data are patchy or heterogeneous [67,77,80,81].

Decision rules for the Combined Margin of Exposure (MOET) are derived directly from the summation of individual risk reciprocals:

$$MOET=\frac{1}{\sum \left(\frac{1}{{MOE}_{i}}\right)}$$

In regulatory practice, if the resulting MOET > 100 (for threshold effects where individual MOEs are based on NOAELs/BMDLs and a standard 100-fold uncertainty factor applies), the combined risk is generally considered acceptable. Conversely, for genotoxic carcinogens, a magnitude of MOET ≥ 10,000 is typically required to conclude a low concern for public health.

A pertinent extension of this metric is the Reference Point Index (RPI) or Point of Departure Index (PODI). Unlike the HI, which sums hazard quotients based on health-based guidance values, the RPI sums the ratios of exposure to a toxicological reference point (RP), such as a NOAEL or BMDL:

$$RPI=\sum (\frac{{Exposure}_{i}}{{RP}_{i}})$$

Under this formulation, the combined risk is essentially the inverse of the Margin of Exposure for the mixture (RPI = 1/MOET). This metric is particularly useful in component-based assessments where harmonized health-based guidance values are lacking for all components, but comparable Points of Departure are available.

Within this scheme, mixtures are generally considered adequately controlled when the RPI remains below 1, equivalently when the MOET exceeds the composite uncertainty factor, typically of the order of 100 for threshold effects. For genotoxic carcinogens, the report noted that MOE values around 10,000 or higher, when derived from a BMDL10, have been used as benchmarks of low concern from a public-health perspective [22].

The practical use of MCR and MOET is tightly coupled to data integration tools. EFSA guidance recommends embedding these metrics within tiered cumulative risk assessment schemes, using them first for broad screening and prioritization, and then for refined, probabilistic evaluations [54,55,82]. Probabilistic exposure tools such as the MCRA toolbox (version 10.2.11.; National Institute for Public Health and the Environment—RIVM, Bilthoven, The Netherlands) demonstrate how integrated exposure and effect databases can be exploited to simulate realistic co-exposure distributions and to explore the sensitivity of MCR and MOET to different uncertainty assumptions [79]. In parallel, curated toxicological repositories such as OpenFoodTox (version 2.0; European Food Safety Authority, Parma, Italy) facilitate the harmonization of PODs and uncertainty factors across large chemical sets, thereby improving consistency in MOET applications [82,83]. Together with advances in computational toxicology and kinetic modelling, these infrastructures are central to the RACEMiC roadmap’s vision of more coherent, cross-sector mixture methodologies [82,84].

Selecting appropriate metrics for a given mixture assessment is therefore not a purely technical choice, but a strategic decision that shapes the interpretability, regulatory relevance and protective capacity of the evaluation. HI remains highly suited to preliminary screening whenever reference values are available and chemicals share at least broadly similar endpoints, offering a conservative first filter for prioritizing mixtures and identifying “hot spots” in food and environmental exposure [54,55,67,96]. RPF and TEQ approaches are preferable when mixtures belong to well-characterized families with common modes of action and robust dose–response datasets, as in the case of dioxins, dioxin-like PCBs and some pesticide groups [25,26,32,68,71]. MCR and MOET, in turn, are best suited to more refined assessments in which harmonized PODs can be established and there is a need either to diagnose the relative importance of mixture components (MCR) or to obtain a single, aggregated margin of exposure (MOET) that can be directly compared with traditional regulatory benchmarks [21,75,77,80,81].

Across all these tools, it is essential to remain explicit about the underlying assumptions—especially dose additivity and the frequent neglect of interaction effects—and about the uncertainties introduced by data gaps, model choices and toxicokinetic variability [69,70,78,98]. Modern frameworks therefore increasingly rely on a portfolio of metrics rather than a single indicator, using HI and MCR for screening, RPF/TEQ where mechanistic coherence permits, and MOET for integrated margins in high-priority scenarios, all embedded in probabilistic and scenario-based modelling platforms such as MCRA and supported by evolving regulatory guidance [79,82,95,96]. This multi-metric, tiered approach offers a scientifically defensible and transparent basis for managing mixture risks in food safety and other high-risk sectors, while remaining flexible enough to incorporate emerging methods and new groups of concern.

### 3.2. Toxicological Models, Synergy and Grouping Strategies

At the toxicological level, consensus has coalesced around Concentration Addition (CA) as the default pragmatic model, supported by evidence that it conservatively predicts mixture toxicity at low doses [5,12,16].

Dose Addition (often referred to as Simple Similar Action or Loewe Additivity) assumes that components behave as dilutions of one another, sharing a common Mode of Action (MOA); individual doses are expressed relative to their effect-producing levels and summed. In contrast, Response Addition (Simple Dissimilar Action or Bliss Independence) assumes that chemicals act via independent MOAs, where the combined effect is calculated probabilistically from individual responses.

$$E_{mix}=1-\prod \left(1-E_{i}\right)$$

While Response Addition is conceptually robust for dissimilar mechanisms, it is seldom used directly in routine human risk assessment because standard reference values (NOAELs, BMDLs) typically lie below the threshold of observable responses.

Thus, CA assumes that components behave as dilutions of each other because they share a common mode of action (MOA) or adverse outcome pathway (AOP); IA assumes statistically independent responses for chemicals acting through different MOAs. CA has become the default regulatory assumption for many cumulative assessments due to its mathematical simplicity, its conservative nature and extensive empirical support, particularly at low effect levels [1,12,13]. Numerous experimental datasets show that CA and IA predictions converge for effect levels below roughly 10–30%, which correspond to typical benchmark response levels used for regulatory PODs [12,15,16]. Moreover, careful analyses indicate that strict MOA homogeneity is not a prerequisite for CA to hold chemicals with ostensibly different MOAs often behave additively when normalized for potency, challenging rigid MOA-based dichotomies [1,5,12,100].

Synergy and antagonism represent deviations from these additivity models, with combined effects exceeding or falling below CA/IA predictions, respectively. Mechanistically, such deviations can arise from toxicokinetic interactions (altered absorption, metabolism, distribution or elimination) or toxicodynamic interactions at the target tissue (competitive binding, receptor cross-talk, changes in signal transduction) [4,101]. Systematic evaluations of pesticide mixtures show that genuine synergy does occur but is quantitatively limited and relatively infrequent under environmentally relevant conditions. Empirical evidence suggests that genuine synergy is rare at environmentally relevant doses; for instance, a landmark review of 194 pesticide mixtures found synergy in only ~7% of cases [20]. Larger meta-analyses of more than 1200 mixture experiments confirm that departures from additivity exceeding a factor of two are rare, especially in the low-dose range relevant for dietary exposure [5,12,16,18,101]. Nonetheless, specific combinations—such as certain triazine, azole and pyrethroid pesticides—have shown synergistic potential, underscoring the need for case-by-case scrutiny [16]. Endocrine-disrupting mixtures can also elicit effects below individual NOAELs under particular experimental designs, although strong synergy under realistic dietary exposures appears uncommon [19,102,103]. Overall, the weight of evidence suggests that dose addition is a sufficiently conservative starting point for most food-relevant mixtures, while acknowledging that synergy cannot be excluded a priori in specific contexts [16,17,18,19,20,21].

Operationalizing mixture assessment at regulatory scale has led to the development of Cumulative Assessment Groups (CAGs), which cluster chemicals into units for joint evaluation based on shared toxicological properties. CAGs may be defined by common target organ or critical effect (e.g., neurotoxicity, thyroid disruption), common MOA/AOP (e.g., organophosphates as acetylcholinesterase inhibitors), shared toxicodynamic outcome (e.g., estrogenic endocrine disruptors), or structural similarity that supports read-across [13,29,93,101,104]. EFSA’s guidance formalizes a tiered, mechanistically informed grouping strategy that prioritizes MOA/AOP evidence, followed by target-organ effects when mechanistic data are incomplete, with chemical class and structure as supporting information [1,27,28,29,93]. In parallel, the concept of common kinetic groups (CKGs) has gained importance to capture toxicokinetic interactions such as enzyme induction or inhibition that may modify internal doses across multiple substances [31,101,104].

In practice, constructing CAGs is constrained by data gaps: for an estimated 60–70% of environmental chemicals, detailed mechanistic information is lacking, many substances act via multiple MOAs, and co-exposure data are sparse, so that CAGs may bundle substances that rarely co-occur in real diets [5,12,80,81]. Recent methodological advances propose dynamic, data-driven grouping using network analysis and machine learning to identify clusters that maximize both toxicological similarity and real-world co-exposure, building on large monitoring and biomonitoring datasets [12,53]. Whatever the grouping strategy, coherent aggregation of hazard and exposure information requires careful selection and harmonization of Points of Departure (PODs). Robust POD selection prioritizes the most sensitive, well-characterized endpoints, giving preference to benchmark dose (BMD) metrics—particularly BMDLs—over NOAEL/LOAEL values because BMD methods exploit the full dose–response curve and provide explicit statistical uncertainty [14,32,74,105,106,107]. Within a CAG, PODs should, as far as possible, be aligned in terms of species, study duration and endpoint definition; marked heterogeneity can necessitate excluding particular studies or substances from quantitative aggregation [25,26,27,31,32,80,81,108].

Where CAG members share a broadly similar MOA but differ in potency, Relative Potency Factors (RPFs) provide a pragmatic way to normalize exposures to a common index chemical, enabling calculation of toxic equivalents. RPF and related Toxic Equivalency Factor (TEF) schemes have been applied to dioxin-like compounds, PAHs, halogenated flame retardants and, more recently, PFAS, with index chemicals chosen based on robust toxicological datasets [32]. For PFAS, RPF approaches explicitly integrate differences in bioaccumulation and toxicokinetic across co-occurring congeners, allowing comparison of aggregate exposures from drinking water and diet against health-protective thresholds and informing remediation priorities where exceedances are likely.

The choice of risk metrics is central to translating CAG-based hazard characterization and exposure estimates into decision-relevant outputs. Regarding metric performance, comparative studies indicate that while HI remains the standard screening tool, MOET offers superior biological interpretability and avoids the obscuring of uncertainty inherent in summing Hazard Quotients [14,21,74,80,81,91,92]. An HI > 1 is often interpreted as a signal of potential concern, with HI ≤ 1 taken as “acceptable”. However, critical analyses have highlighted several limitations: HI assumes perfect dose addition, aggregates across potentially disparate endpoints, offers no explicit probabilistic or biological interpretation of the threshold at 1, and obscures uncertainty in both exposure and reference values [14,20,74,76,86,87]. Furthermore, reference values themselves are derived from heterogeneous assessment factors and data quality, so HI is best regarded as a screening-level indicator rather than a predictor of population-level effect, particularly when used without explicit uncertainty analysis [20,21,80,81].

Margin-of-Exposure-based metrics, notably the combined Margin of Exposure Total (MOET or nMOET), have gained prominence as more informative alternatives for refined, component-based cumulative assessments [13,74,77,91,92]. For single substances, the MOE links exposure directly to a benchmark dose associated with a specified response level (often 10%), and its interpretation is straightforward: a MOE of 100 implies that the current exposure is 100-fold below the dose associated with the benchmark effect. For mixtures, MOET is calculated as the reciprocal of the sum of reciprocals of component MOEs, reflecting dose-addition principles [13,74]. This formulation preserves a clear probabilistic and biological meaning, aligns naturally with default uncertainty factors (e.g., MOE ≥ 100 for threshold toxicants; ≥10,000 for genotoxic carcinogens), and facilitates identification of “risk drivers” through contribution analysis of individual MOEs [74,76,77,91,92,109]. Comparative studies indicate that MOE/MOET metrics perform at least as well as, and often better than, HI in capturing apical toxicological outcomes across diverse environmental mixture datasets [74,76,77]. Nonetheless, MOET thresholds remain pragmatic guides rather than absolute safety lines; their interpretation must explicitly account for cumulative uncertainty [8,20,21,87,91,92].

Case studies illustrate both the feasibility and constraints of implementing these concepts in routine risk assessment. EFSA’s cumulative assessments for triazole fungicides and other pesticide CAGs have applied dose addition, BMD-based PODs, RPF scaling and MOET-type metrics to large monitoring datasets, highlighting typical challenges such as data gaps, aggregate exposure uncertainty and the dominance of a few compounds in overall risk [1,12,13,27,28,29,93,108]. Similar patterns emerge in cumulative dietary exposure analyses for endocrine disruptors and other priority groups, where a small subset of chemicals often contributes disproportionately to HI or MOET, guiding risk-management efforts toward targeted substitution or control of these “risk drivers” [74,76,109]. In non-dietary contexts, such as occupational pesticide use, mixture exposure is characterized by route-specific patterns and intermittent high-exposure “mixing events”, requiring tailored study designs and integration of job-specific scenarios [31]. Incorporating human biomonitoring and epidemiological data into these assessments strengthens the linkage between external exposure metrics and internal dose–response relationships, enabling validation of model predictions at realistic exposure levels [14,53,74,87,107].

To support clarity and facilitate navigation through the methodological framework, Table 1 summarises the key parameters and metrics used in toxicity evaluation and chemical mixture risk assessment, together with their role and main interpretation aspects.

## 4. Regulatory Frameworks for Cumulative Risk Assessment

Over the last decade and a half, regulatory approaches to chemical mixtures have evolved from largely conceptual discussions into operational frameworks embedded in major food safety and public health agencies. The European Food Safety Authority (EFSA), the U.S. Environmental Protection Agency (EPA), and joint WHO/FAO initiatives now share a common methodological backbone built on tiered assessment, mechanism- or effect-based grouping of chemicals, and dose addition as the default model for substances with similar or overlapping modes of action [1,23], Within this shared architecture, the agencies differ in scope, data infrastructure, and degree of legal enforceability, leading to complementary but not yet fully harmonized implementations of cumulative risk assessment (CRA) for pesticides and other food-borne xenobiotics [86,87,91,92].

### 4.1. European Food Safety Authority (EFSA)

EFSA’s 2013 international review synthesized these developments into a generic tiered framework for combined exposure and risk, spanning qualitative tier-0 screens to fully probabilistic tier-3 assessments. Within this structure, two overarching hazard strategies are distinguished: whole-mixture assessment when adequate toxicity data exist for the tested or a sufficiently similar mixture, and component-based assessment when dose–response information is available for individual substances. Component-based assessments rely on defining Cumulative Assessment Groups or Assessment Groups (CAGs/AGs), ideally based on shared target organs and modes or mechanisms of action, to delineate which chemicals are combined in a given cumulative evaluation [22]. Methodologically, two overarching strategies are distinguished depending on data availability. The Whole Mixture Approach treats the entire mixture as a single entity—accounting for unidentified components and inherent interactions—and is applied when toxicological data exists for the specific mixture or a virtually identical one. Conversely, the Component-Based Approach is employed when toxicity and exposure data are available for individual constituents, relying on their grouping into Cumulative Assessment Groups (CAGs) based on weight-of-evidence, dosimetry, or mechanistic data.

EFSA’s 2019 guidance [1] represents one of the most comprehensive attempts to operationalize CRA in routine regulatory practice. It formalizes a tiered scheme that combines component-based and whole-mixture approaches, using dose addition as the default assumption for chemicals acting on a common target or effect, while allowing response addition where mechanisms are clearly dissimilar. A central innovation is the concept of Cumulative Assessment Groups (CAGs), defined on the basis of shared toxicological endpoints or mechanistic concordance, which allows transparent grouping of active substances for joint evaluation [93].

Early EFSA work on pesticides laid the foundations for these CAGs, particularly for compounds affecting the nervous system and thyroid, demonstrating that such groupings could be implemented in routine opinions [27,28]. Subsequent applications extended CRA to more specific developmental outcomes, such as craniofacial malformations, and to complex exposure scenarios, thereby illustrating both scientific feasibility and regulatory relevance [29,31].

EFSA’s framework is tightly integrated into the EU risk assessment cycle and supported by rich dietary exposure databases and probabilistic tools. Recent initiatives, such as the RACEMiC roadmap, further emphasize uncertainty analysis, probabilistic modelling, and transparent communication of cumulative risks to decision-makers and the public [82]. This combination of structured grouping principles, quantitative methodology, and legal embedding makes EFSA’s approach a reference point for CRA in food-related mixtures.

### 4.2. U.S. Environmental Protection Agency (EPA)

The U.S. EPA has pioneered mixture assessment within pesticide regulation, particularly through its work on organophosphate insecticides. Its cumulative risk paradigm is firmly rooted in “common mechanism groups”, in which pesticides are grouped by a shared mode of action and assessed collectively [25,26,32]. Within these groups, the EPA typically combines exposures using Relative Potency Factors (RPFs) to normalize doses to an index compound, and often expresses combined risk via hazard indices, provided that the toxicological endpoint, test species, and study conditions are sufficiently comparable to support relative potency assumptions [80,81].

The U.S. EPA cumulative-risk framework operates as a predominantly component-based, dose-additive system that can be extended to interaction-adjusted indices. Whole-mixture assessment is applied when mixture-specific reference doses, reference concentrations or cancer slope factors are available, whereas component-based assessments group chemicals into assessment groups defined by common sources, target organs or modes of action. The framework explicitly encourages integration of multiple routes and timings of exposure, use of internal-dose measurements to reconcile aggregate exposure across routes, and the application of statistical tools such as categorical regression to model multi-endpoint responses, all underpinned by problem formulation and explicit analysis of variability and uncertainty [22].

This approach is strongly quantitative and data-driven, focusing on pesticide families with well-characterized hazard and exposure profiles. Over time, the EPA has incorporated more sophisticated modelling components, including probabilistic dietary and residential exposure assessments and the use of high-throughput screening and exposure models (e.g., ToxCast and ExpoCast) to inform grouping and prioritization [24]. As a result, EPA’s framework is one of the most operationalized in terms of completed cumulative assessments but remains largely restricted to pesticides and other domains where mechanistic evidence and exposure data are robust. This domain specificity highlights both the strengths and the boundaries of mechanism-centered, RPF-based CRA.

### 4.3. WHO/FAO International Frameworks

At the international level, WHO and FAO have assumed a primarily normative and facilitative role, aiming to harmonize terminology, core principles, and practical approaches to CRA across countries with very heterogeneous regulatory capacities. The FAO/WHO 2019 Framework and subsequent guidance documents propose a proportionate stepwise process for combined exposure and risk assessment, anchored in explicit problem formulation, tiered exposure characterization, and transparent documentation of uncertainties [23].

These documents endorse dose addition as the default model for chemicals with similar modes or mechanisms of action, in line with EFSA and EPA practice, while acknowledging the need for pragmatic simplifications in data-poor settings [80,81]. A key feature is their resource-sensitive orientation: the guidance explicitly distinguishes between basic screening tiers—suitable for low- and middle-income countries with limited data and modelling infrastructure—and more advanced tiers that can be implemented where national systems and datasets permit. Through the Codex Alimentarius system and related capacity-building initiatives, WHO/FAO seeks to embed cumulative assessment principles into international food safety standards and trade frameworks, promoting gradual convergence without imposing unrealistic technical demands [7,23].

### 4.4. Convergence and Divergence

Despite differing mandates, EFSA, EPA, and WHO/FAO converge on core methodological pillars, most notably the endorsement of tiered assessment schemes and mechanism-based grouping [1,23]. Grouping strategies are consistently based on shared mechanisms or health outcomes, whether framed as CAGs, common mechanism groups, or effect-based clusters—reflecting a broad consensus that mixture assessment should be biologically informed rather than purely structural. Dose addition is universally recognized as the default model for mixtures of similarly acting chemicals, with response addition reserved for clearly independent modes of action [1,32].

Where the frameworks diverge is in breadth of application, legal embedding, and depth of quantitative implementation. EFSA has integrated CRA into its routine regulatory cycle for pesticides and is extending it to other contaminant domains, underpinned by comprehensive European dietary datasets and probabilistic tools [82]. EPA’s framework is narrower but particularly advanced for specific pesticide classes, leveraging RPFs, hazard indices, and high-throughput data streams [24,25,26]. WHO/FAO, in contrast, emphasizes globally applicable principles and scalable methods to ensure that countries with limited infrastructure can still implement scientifically defensible CRA, even if at simplified tiers [23].

These differences also reflect distinct decision contexts and risk-management cultures. EFSA operates within a stringent EU legal framework requiring precautionary protection of vulnerable groups, which encourages detailed uncertainty analysis and probabilistic modelling [91,92]. EPA’s decisions are similarly grounded in statutory mandates but often navigate different risk-benefit and economic considerations. WHO/FAO, meanwhile, must provide guidance that can be adapted to a wide diversity of national legal and social contexts, making flexibility and proportionality central design features [86,87].

Collectively, these trajectories illustrate a definitive paradigm shift from single-chemical assessments toward cumulative frameworks (Table 2). CRA has moved from being primarily a research topic to becoming an operational regulatory tool, especially in the pesticide domain [1,23]. This evolution has been driven both by advances in mixture toxicology and by growing recognition that background co-exposures can meaningfully influence health risks, particularly in sensitive populations.

From a regulatory perspective, the three major international frameworks show a high degree of conceptual consistency, as they are all grounded in tiered cumulative assessment approaches, broadly converge on dose addition as the default assumption for chemicals with similar or overlapping effects, and rely on metrics such as HI, MOE/MOET and potency-based approaches (RPF/TEQ) when mechanistic justification is available. However, important differences remain in terms of scope, legal status and operational implementation. EFSA provides a legally binding and probabilistically advanced framework closely integrated with European food safety legislation and large exposure databases; the U.S. EPA framework is strongly rooted in pesticide legislation under the Food Quality Protection Act (FQPA), with particular emphasis on RPF-based approaches and cumulative group definitions; whereas the FAO/WHO system offers globally applicable but non-binding guidance designed to support harmonisation and capacity building across regions. Importantly, these frameworks are not merely academic constructs: they are directly or indirectly linked to regulatory decision-making, including product approval, tolerance setting, re-evaluation of substances and international risk management through Codex-related processes [7,24,82].

Nevertheless, substantial challenges remain before a fully coherent, globally harmonized system for mixture risk assessment can be realized. Differences in data infrastructure, mechanistic understanding, and legal obligations still lead to uneven implementation across agencies and regions. EFSA’s legally embedded, data-rich framework, EPA’s domain-specific but operationalized pesticide assessments, and WHO/FAO’s resource-sensitive global guidance exemplify complementary pieces of an emerging international architecture rather than a single unified system [7,24,82].

Recent initiatives—including EFSA’s RACEMiC roadmap, EPA’s NexGen-style efforts to integrate high-throughput and computational toxicology into decision-making, and the WHO/FAO Framework for Combined Exposure—signal an ongoing scientific and institutional convergence. In parallel, coordination platforms such as OECD working parties and Codex committees are facilitating gradual alignment of terminology, data standards, and modelling practices [23,80,81]. If sustained, this convergence could support more consistent protection of public health against cumulative exposures, while enabling regulators to better manage uncertainty and communicate mixture risks in a transparent, evidence-based manner [86,87,91,92].

## 5. Exposure Data Streams Supporting Mixture Risk Assessment

### 5.1. Total Diet Studies (TDS): Design and Best Practices

Total Diet Studies (TDS) are now widely regarded as a core public health tool for assessing chronic dietary exposure to chemicals, including both contaminants and nutrients, under realistic conditions of consumption. By focusing on foods “as eaten” and integrating them according to actual dietary patterns, TDS provide exposure estimates that are generally more representative and policy-relevant than those derived from conventional monitoring of raw commodities [34]. Historically, the concept emerged in the United States in the late 1950s and has since expanded globally as a standard instrument for characterizing population exposure to a wide range of chemicals in the diet [35,41,42].

Over the last decade, substantial efforts have been devoted to methodological harmonization. The joint guidance developed by EFSA, FAO, and WHO provides the conceptual foundation of modern TDS, defining key elements such as food list construction, sampling, preparation, pooling and analytical requirements [34]. These principles have been operationalized and refined in international initiatives such as the TDS-Exposure project, which has contributed detailed experience on food grouping, composite design and data handling across European countries [36,37]. National implementations further show that alignment of TDS design with specific policy questions and risk-management needs is essential for ensuring sustainable financing and long-term continuity [41,42,110].

Methodological design rests on three pillars: (i) selection of representative foods that collectively cover the vast majority of dietary intake, (ii) preparation of foods as consumed at the table, and (iii) aggregation (pooling) of similar foods into composite samples for chemical analysis [34]. In practice, food lists are derived from individual consumption surveys and are generally constructed to cover at least 85–90% of total energy or mass intake, depending on the diversity of national diets [34,38]. These lists are typically organized into the order of 100–300 TDS food groups or composites, allowing a feasible balance between analytical workload and dietary representativeness [37]. Because food markets and dietary patterns evolve, periodic updating of food lists is indispensable to maintain ≥90% coverage and to ensure that new products or consumption trends (e.g., plant-based alternatives, convenience foods) are captured [38,41,42].

Detailed national examples illustrate how these design concepts are implemented. In a French children’s TDS, 309 composite samples were prepared, each constructed as a pool of 12 subsamples of the same food collected over a one-year period, varying in brand, outlet and preparation method; this strategy captured both temporal and market-related variability while keeping the number of analytical determinations manageable [39]. In Portugal, the inclusion of “regional seasonal” samples was used to account explicitly for seasonal and geographical variability in chemical levels or consumption patterns, demonstrating how TDS can be tailored to specific climatic and cultural contexts [37]. These examples highlight the central role of robust consumption data and careful food aggregation rules in building a food list that is both comprehensive and operational.

EFSA’s 2013 review already stressed that simple summation of exposures across chemicals neglects correlations in their occurrence, which can bias estimates of cumulative risk. To better characterize realistic co-exposure, the report highlighted the importance of multi-analyte analytical strategies and Total Diet Studies that measure several residues or congeners in the same composite samples, providing joint occurrence data that can be coupled with models such as ACROPOLIS to explore occurrence–exposure correlations [22].

Sampling strategies must reflect the diversity of the food supply to ensure that composite samples are representative of actual exposure. Guidance from WHO/FAO recommends that in urban settings, foods be collected from multiple outlets and brands, typically 5–10 different retail locations per food group, while also considering different production systems where relevant (e.g., conventional vs. organic) [34]. Combining purchases across chains, neighborhood stores and, where appropriate, direct sales from producers reduces selection bias and better represents the range of products available to consumers [37,39].

Food preparation is another critical step, as it aims to mimic domestic culinary practices such as washing, peeling, trimming and cooking, thereby reflecting the actual concentration of chemicals at the point of consumption [34,111]. Processing can markedly increase or decrease contaminant concentrations, for example, by water loss during cooking, removal with peel, or formation of processing contaminants. Consequently, TDS protocols must document in detail the applied recipes, cooking times, temperatures and preparation methods, ideally based on population-level dietary surveys and culinary information [39,111]. Harmonized, well-documented home-style preparation SOPs are essential for comparability across time and between countries [34].

Analytically, TDS pose specific challenges because composite samples combine several foods and often encompass complex matrices in which multiple chemical classes must be quantified simultaneously. This demands highly sensitive, accurate and robust methods, frequently based on multi-element or advanced multi-analyte techniques (e.g., ICP-MS, LC-MS/MS) capable of quantifying diverse chemical classes within complex matrices [40,41,42]. Advances in multi-residue analytical platforms continue to improve coverage and throughput but must be accompanied by rigorous method validation and ongoing quality control to ensure comparability across matrices and over time [112]. Key technical issues include managing matrix effects, ensuring storage stability of analytes in composite samples, and the limited availability of appropriate certified reference materials for complex food matrices [34,112].

Handling left-censored data remains a critical source of uncertainty. Standard approaches involve substitution (e.g., lower-bound, upper-bound) or distribution-based imputation in TDS. Because a large proportion of measurements—often >50% for some analytes—may fall below LOQ, exposure estimates can be sensitive to the chosen substitution or modelling approach. Common pragmatic options include assigning values of LOQ/2 or LOQ/√2, while more advanced analyses may employ distribution-based or multiple-imputation methods [34]. The choice should be informed by the proportion of censored results, the toxicological relevance of potential over- or underestimation, and the broader purpose of the assessment, especially when TDS outputs feed into cumulative or combined risk assessments.

TDS results are translated into exposure estimates by combining concentration data in composite samples with individual or group-level food consumption distributions. Deterministic approaches, which apply point estimates (e.g., mean or high-percentile concentrations and intakes), are straightforward and transparent, but may not capture the full variability within the population [34]. Probabilistic methods, by contrast, integrate the distributions of concentration and consumption, providing a more realistic characterization of inter-individual variability and uncertainty, at the cost of increased data demands and methodological complexity [34]. Health risks are then evaluated by comparing chronic exposure estimates with health-based guidance values (HBGV), such as tolerable daily or weekly intakes (TDI, TWI) and upper intake levels (UL), or, for substances without an identified threshold, by calculating margins of exposure (MOE) or combined margins of exposure (MOET) [40]. Explicit consideration of vulnerable subgroups—for example infants, young children or high consumers of specific food categories—is essential, as their exposure patterns and toxicological sensitivities can differ markedly from the general population [39,111].

Overall, experience indicates that TDS provides the most reliable and cost-effective basis for assessing chronic dietary exposure at the population level, often with lower uncertainty than exposure assessments based solely on standard monitoring data [43]. However, the very design features that make TDS efficient also entail limitations. The pooling of foods into composites leads to a loss of information about the variability of concentrations between individual foods, brands or batches, and makes it impossible to identify highly contaminated individual products or outliers [44,45]. In addition, aggregation decisions (e.g., which foods are grouped together) and the representativeness of sampling can influence risk estimates, underscoring the importance of transparent documentation and sensitivity analyses [34].

Several key recommendations emerge from recent applications. First, integration of TDS data with national food monitoring programs is strongly advised to exploit the complementary strengths of each source: TDS offers realistic chronic exposure estimates, while monitoring data preserve product-level variability and support enforcement and source attribution [43,44,45]. Second, harmonization of reporting formats is crucial to enable cross-country comparison and pooled analyses. This includes agreement on minimal reporting elements such as FoodEx2-based coding and pooling schemes, age- and sex-stratified exposure estimates, and harmonized descriptive statistics for concentrations and intakes [34,41,42]. Third, food lists and composite structures should be periodically revised to maintain high coverage of current diets and to incorporate emerging food categories, thereby ensuring continued relevance of TDS outputs for risk assessment and policy [38,41,42].

Looking ahead, the strategic value of TDS is likely to increase as new classes of dietary contaminants emerge (e.g., nanomaterials, microplastics) and as integration with human biomonitoring (HBM) and advanced exposure modelling becomes more routine. Linking TDS data with HBM can help validate exposure models, refine toxicokinetic assumptions and improve the interpretation of biomarker levels at the population level. At the same time, advances in data science and artificial intelligence—for example, predictive models of contamination patterns across food chains or tools for optimizing sampling designs—offer opportunities to enhance efficiency and tailor TDS to evolving policy priorities [41,42,112]. Realizing this potential will require sustained multi-stakeholder collaboration, robust data infrastructures and clear alignment of TDS programs with national and international risk-management agendas [36,110].

### 5.2. Human Biomonitoring (HBM) of Dietary Origin

Monitoring chemical mixtures in foods increasingly requires moving beyond estimates of external exposure to directly measuring internal human doses. Human biomonitoring (HBM) provides this connection by quantifying parent compounds, metabolites or adducts in biological matrices, thereby capturing real-life exposure from all routes and sources, including diet, and the modifying role of toxicokinetic [48,52,103]. Coordinated initiatives such as HBM4EU, EFSA’s harmonized frameworks and WHO guidance have consolidated HBM as a crucial complement to occurrence-based exposure assessments, including Total Diet Studies (TDS), particularly when HBM data are interpreted alongside food-consumption information and mixture-risk metrics such as the Hazard Index (HI) or the Margin of Exposure for mixtures (MOET) [47,50,53,59]. In this integrated perspective, HBM translates food-chemical occurrence into evidence-based indicators of internal exposure and potential health risk, reducing uncertainty in mixture risk assessments.

Applied to diet, HBM is embedded in the broader concept of the dietary exposome, i.e., the ensemble of exogenous and endogenous chemicals that enter the body through food and interact with biological systems over the life course [10,11,113,114,115]. Serum, urine, breast milk and other matrices capture both persistent and short-lived compounds and thus complement TDS, which estimate external intakes from food and drinking water [6,103]. The European exposure-science strategy 2020–2030 explicitly frames HBM as part of a 21st-century toolbox that integrates analytical chemistry, omics, and exposure modelling within an exposomic paradigm [48,50]. Nutritional metabolomics and untargeted exposomic workflows further extend this scope by enabling simultaneous measurement of food-derived metabolites, environmental contaminants and endogenous response markers, revealing dietary signatures and co-exposure patterns that can be linked back to foods or dietary patterns [115,116,117]. WHO and regional European guidance emphasize that diet-focused HBM must rely on standardized sampling, robust ethical protocols and harmonized QA/QC to ensure cross-population comparability and policy relevance [52].

The selection of effective dietary biomarkers is central to the interpretability of HBM data. Beyond analytical sensitivity and specificity, biomarkers must be biologically and epidemiologically meaningful, i.e., they should reflect the relevant exposure window, allow linkage to dietary sources and support dose–response assessment [64,66,114]. Time window and representativeness are key: short-lived chemicals such as phthalates or bisphenols, with urinary half-lives of hours, require sampling strategies that capture within-person variability, including repeated or first-morning urine samples, or the use of metabolites with longer integration windows [60,62,103]. In contrast, persistent pollutants such as many PFAS and some metals accumulate in long-lived matrices (e.g., blood, hair) and provide stable indicators of long-term exposure [54,55,56,57,118]. Reproducibility metrics illustrate these issues: alkylresorcinols as biomarkers of whole-grain intake show intra-class correlation coefficients around 0.38–0.74 over 2–36 months, which is acceptable but still imposes constraints on statistical power and on the interpretation of diet–disease associations [114]. Across EFSA and HBM4EU, converging guidance highlights four core criteria for dietary biomarkers—sensitivity at low environmental levels, specificity for relevant exposure routes (ideally diet), temporal stability within the intended window, and inter-individual reproducibility—along with explicit characterization of uncertainty and comparability to reference populations when used in mixture evaluations [52,116].

Case studies for specific chemical families exemplify both the strengths and the limitations of dietary HBM. For per- and polyfluoroalkyl substances (PFAS), HBM data demonstrate high persistence and a strong linkage to diet in many populations. Serum half-lives vary widely, ranging from weeks (e.g., PFBS) to years (e.g., PFOS, PFHxS), thus providing long integration windows and relatively high dietary specificity, especially where drinking water and certain foods (fish, eggs, offal) are dominant contributors [53,54,55,56,57,103]. Under HBM4EU, adolescent reference values (RVs) were derived for 12 PFAS; European HBM data indicated that around 14.3% of teenagers exceeded a serum sum of 6.9 μg/L, a level corresponding to EFSA’s TWI of 4.4 ng/kg bw per week, underscoring the public health relevance of dietary exposure [54,55,58]. Syntheses of PFAS exposure confirm that diet is often the main pathway in the general population, particularly where drinking water is controlled, and that serum levels can be quantitatively related to long-term intakes using physiologically based pharmacokinetic (PBPK) models [6,9,25,26,59].

To explicitly account for absorption, distribution, and body burden, Internal Hazard Indices (HI_int_) can be derived using toxicokinetically corrected metrics. This involves calculating Internal Hazard Quotients (IHQ_i_) defined as:

$${IHQ}_{i}=\frac{{Internal\;Exposure}_{i}}{{RV}_{i}}$$

Alternatively, the Internal Dose Toxic Unit (IDTU) approach sums the ratios of internal concentrations (C_i_) normalized by Critical Body Residues (CBR_i_) or bioaccumulation factors (BAF_i_):

$$ID\sum TU=\sum \left(C_{i}\frac{{BAF}_{i}}{{CBR}_{i}}\right)$$

These internal metrics refine the assessment by integrating the net result of bioavailability and metabolic interactions, offering a more proximal indicator of tissue-level risk than external dietary estimates.

For phthalates and bisphenols, short biological half-lives create challenges for interpretability. Single spot urine samples may poorly represent habitual exposure, especially when product use and diet vary day to day [60,62]. Nonetheless, HBM4EU results show that HBM guidance values (HBM-GVs) for several phthalates and substitutes are exceeded in specific subgroups, particularly children and adolescents, and that mixture-based assessments can reveal anti-androgenic risks missed by single-substance approaches—around 17% of children/adolescents may experience anti-androgenic mixture risks from phthalates at current exposure levels [61,62]. EFSA’s recent review of bisphenol A (BPA) illustrates how urinary biomarkers can be used to relate internal exposure to health-based guidance values: a very low TDI of 0.2 ng/kg bw per day, based on immune (Th17) effects, implies that even modest urinary concentrations can correspond to exceedances of health-based limits for some consumers [119]. Here, diet (e.g., canned foods, food contact materials) and non-dietary sources (e.g., thermal paper, consumer products) both contribute, underscoring the need for careful attribution of exposure pathways in mixture assessments [60,62].

Mycotoxins provide an example of high dietary specificity combined with variable exposure windows. Urinary biomarkers of ochratoxin A, deoxynivalenol and fumonisins are increasingly used to estimate chronic intake and to identify sub-populations at risk, particularly among cereal consumers [8,63,64,65]. Biomarkers based on protein adducts, such as aflatoxin–albumin or hemoglobin adducts, integrate exposure over weeks to months and are thus suited to chronic-risk characterization, but regional heterogeneity, seasonality and co-occurrence of multiple mycotoxins complicate cross-country comparisons and mixture evaluations [8,103]. Overall, these chemical-family examples illustrate how dietary HBM can address both longstanding and emerging contaminants, and how family-specific toxicokinetics shape the choice of biomarker, sampling strategy and mixture-risk modelling approach [47,59].

Integrating HBM with consumption data, TDS and PBPK modelling is pivotal for transforming biomarker concentrations into quantitative intake estimates suitable for mixture-risk assessment. In PFAS case studies, PBPK models show that diet is often the dominant contributor to internal exposure, but that other sources (e.g., drinking water, consumer products) can be important in specific subgroups; modelled internal exposures typically align with, but may be somewhat lower than, measured serum concentrations, reflecting uncertainties in parameters and unaccounted sources [6,56,57,59]. For PFOA, a study in women showed that personal-care products could exceed dietary contributions in some individuals, highlighting source-specific heterogeneity even when diet is the main pathway at population level [120]. At the program level, comprehensive national strategies combining market monitoring, TDS and HBM have been proposed and implemented to characterize exposure distributions and to benchmark them against health-based guidance values in a consistent way [46,53]. EFSA’s methodology explicitly recommends the use of PBPK modelling and reverse dosimetry to convert biomarker levels into equivalent daily intakes that can be compared with TDIs, TWIs or BMDLs and integrated into HI or MOET calculations [50,54,55,59].

Robust reporting and harmonization are essential to ensure that dietary HBM data can be pooled, compared to and used in regulatory decision-making for chemical mixtures. The HBM4EU project highlighted harmonized QA/QC schemes, inter-laboratory comparability, and the availability of suitable reference materials as prerequisites for reliable data [47,49,121]. Building on this, the “Minimum Information Requirements” (MIR) proposed by Zare Jeddi et al. specify key elements that should be reported from study design through publication—population descriptors, sampling windows, analytical methods and performance, and left-censor handling—to maximize data quality, accessibility and interpretability [50,51,122]. WHO guidance complements MIR with explicit recommendations on ethics, participant feedback (including communication of individual results in cases of concern) and laboratory QA/QC [52]. EFSA and HBM4EU also point to persistent deficits in contextual metadata—such as mixture co-exposures, diet and lifestyle confounders, and socio-economic variables—as major obstacles to mixture-aware analyses and to the identification of vulnerable groups [53,61,62,114].

Despite rapid progress, important knowledge gaps remain. These include the scarcity of longitudinal datasets across life stages, limited integration of non-persistent compounds in routine monitoring, under-representation of vulnerable groups such as children and pregnant women, and the lack of validated biomarkers for emerging hazards (e.g., nanomaterials, microplastics) and for mixture-aware exposure or effect indicators [49,50,52,66,117]. Nonetheless, dietary HBM is clearly shifting from exploratory pilot projects towards policy-focused surveillance, providing direct human evidence for the evaluation of mixture-risk methods such as HI and MOET and for the assessment of risk-management effectiveness [47,53,59,121]. Coordinated implementation of MIR, sustained EFSA–WHO collaboration and further development and validation of PBPK models for diverse chemical families will be crucial to fully exploit the potential of HBM in integrated exposure assessments [51,52]. The policy relevance of this approach is already evident: HBM4EU results are directly supporting implementation of the EU Chemicals Strategy for Sustainability and the Zero Pollution Action Plan, demonstrating how HBM data can be used to track progress towards exposure reduction goals and to evaluate the effectiveness of regulatory actions targeting dietary mixtures of xenobiotics [123].

These opportunities and remaining challenges are revisited in Section 6, where their implications for uncertainty management, regulatory implementation and future research priorities are considered.

## 6. Discussion: Performance, Best Practices, Uncertainty and Challenges

### 6.1. Best Practices for Implementing Mixture Risk Assessment

Across these applications, recurrent errors can undermine the robustness of mixture assessments. These include unjustified extrapolation of synergy beyond narrowly defined experimental conditions, misclassification of MOA similarity leading to inappropriate selection of CA or IA, inconsistent or poorly documented grouping criteria, and neglect of covariance in co-exposure patterns, even though source, use and physiological correlations between components are frequent [20,74,76,80,81,109]. A particularly pervasive issue is the categorical interpretation of HI < 1 or MOET above default thresholds as evidence that a mixture is “safe”, without adequately conveying residual uncertainty, susceptible subpopulations or the conditional nature of model assumptions [21,85,86,87]. In communication with risk managers and the public, failing to explain these nuances can erode trust and obscure the precautionary or conservative elements embedded in mixture assessments [87,89,90].

Best practices dictate a tiered approach rooted in rigorous problem formulation, where effort is allocated proportionally to risk [1,13,14,27,28,80,81,93]. Tiered assessment frameworks allocate effort proportionally to the level of concern, starting with screening-level HI or MOET calculations based on deterministic exposure estimates and progressively refining exposure (e.g., probabilistic models, individual-level consumption data) and hazard characterization (e.g., BMD-based PODs, RPF scaling, kinetic modelling) only when indicated [31,74,76]. Within this paradigm, harmonized BMDL-based PODs, mechanistically and kinetically informed CAGs/CKGs, and explicit handling of co-exposure correlations and susceptible groups are crucial [5,12,32,101,104]. Whenever possible, model predictions should be checked against empirical mixture toxicity data at realistic exposure levels, and uncertainty should be characterized both qualitatively and quantitatively, including sensitivity analysis of key assumptions [14,77,80,81,89].

### 6.2. Applicability and Prioritisation of Cumulative Risk Assessment Methodologies

Different methodologies in mixture risk assessment do not compete but fulfil complementary roles within a tiered framework. In practice, HI provides a conservative screening signal when health-based guidance values exist, while MOET enables a more refined interpretation based on PODs without compounding assessment factors. When strong mechanistic evidence supports similar modes of action, RPF/TEQ approaches allow potency-adjusted aggregation. MCR does not quantify risk itself but is highly informative to understand whether cumulative risk is driven by one or multiple components, guiding monitoring priorities and regulatory focus. Finally, probabilistic tools and HBM–PBPK integration become particularly valuable when policy relevance depends on explicitly addressing variability, uncertainty and internal dose. Table 3 summarises when each approach is most applicable and when refinement becomes necessary, providing practical guidance for researchers and risk assessors.

### 6.3. Uncertainty in Current Mixture Risk Assessment

Uncertainty permeates mixture assessment at three interconnected levels: parameter (data quality), model (additivity assumptions), and scenario (framing of the exposome) [20,87].

Across the frameworks it reviewed, EFSA concluded in 2013 that uncertainty analysis is integral to each tier of mixture risk assessment rather than an optional add-on. Depending on data availability, the report recommended qualitative, semi-quantitative or fully probabilistic approaches, with explicit identification of major data gaps, the magnitude of parameter and model uncertainty, and the implications for risk-management decisions. Emphasis was placed on problem formulation—defining assessment groups, metrics and tier level in light of regulatory questions such as cumulative assessment of pesticides under Regulation (EC) No. 396/2005—so that mixture assessments remain both scientifically robust and decision-relevant [22].

Model uncertainty reflects the selection of metrics (e.g., HI vs. MOET vs. RPF/TEQ), mixture assumptions (dose vs. response addition, neglect or inclusion of interactions) and cross-species or route extrapolations embedded in PBPK models and default assessment factors [21,85,86,87]. Scenario uncertainty refers to how the problem is framed—selection of CAGs, time windows (acute vs. chronic, life-stage specific), population subgroups and co-exposure contexts—which effectively determines which parts of the exposome are brought into the assessment and which remain outside its boundaries [20,86,87]. Together, these layers motivate explicit, structured uncertainty analysis as a prerequisite for robust interpretation and risk management in mixture assessments.

To move from qualitative acknowledgment to quantitative characterization, mixture frameworks increasingly rely on local and global sensitivity analyses. These tools identify which parameters (e.g., occurrence in key commodities, high-percentile consumption, specific PODs or TK parameters) drive variability in HI or MOET, and which uncertainties most affect decision-relevant outputs [87]. In practice, such analyses consistently show that cumulative risk is often dominated by a relatively small subset of substances—“risk drivers”—even when hundreds of components are formally included [20,67,87]. This pattern has been demonstrated in cumulative dietary assessments for thyroid-active pesticides, where a limited number of compounds explain most of the group risk [124], and in co-exposure to mycotoxins, where a few toxins drive combined MOE deficits [67]. Identifying such drivers is crucial for prioritizing monitoring, refining toxicological datasets, and targeting risk-management options, while acknowledging that tail contributions from many low-level co-exposures remain a structural source of residual uncertainty [20,21].

Under high or structurally irreducible uncertainty—for instance, when interactions cannot be ruled out or when data gaps are systematic—the precautionary principle provides a legitimate basis for conservative decisions and for revisiting assessment factors in mixture settings [21,85,86,91,92]. Organizations specialized in risk sciences stress that such precaution must be explicit, time-bound and linked to targeted data-generation plans rather than treated as a permanent substitute for analysis [21].

Delivering credible mixture assessments also depends on documentation standards and traceability. Recording of all key modelling choices—problem formulation, grouping criteria, inclusion/exclusion rules, POD selection, metric choice, exposure scenarios and uncertainty handling—facilitates internal QA/QC, peer review and subsequent updates [87,89]. Harmonized templates and uncertainty catalogues, aligned across agencies and projects, support comparability between assessments and reduce duplication of effort [89].

Despite these advances, important methodological and practical constraints remain that shape how mixture risk assessment can currently be implemented in real-world settings.

Screening metrics such as the Hazard Index may still be interpreted as binary outcomes, potentially masking residual uncertainty and susceptible subgroups. MOE/MOET approaches depend critically on the availability and harmonization of robust PODs; potency-based aggregation requires convincing evidence of mechanistic similarity; and diagnostic tools such as MCR, while extremely informative for prioritisation, do not provide an absolute measure of health risk. In parallel, applied research consistently reveals structural challenges, including limited longitudinal datasets, insufficient coverage of non-persistent compounds, under-representation of vulnerable populations, and a scarcity of validated biomarkers or mixture-sensitive indicators. These elements continue to constrain the interpretability, generalizability and regulatory usability of mixture assessments in practice [19,67,85,86,87].

At the same time, several developments clearly act as drivers of methodological optimisation: increasing integration of Total Diet Studies and Human Biomonitoring, advances in PBPK modelling, wider adoption of probabilistic approaches to variability and uncertainty, and the emerging use of large-scale data analytics to characterize co-exposure patterns. However, persistent bottlenecks remain, particularly the need for reliable mixture-specific biomarkers, the empirical difficulty of characterizing interactions under realistic exposure conditions, structural uncertainty linked to problem formulation, and uneven regulatory capacity and data infrastructure across regions. Together, these factors explain both the significant methodological progress achieved to date and the barriers that still limit full operationalization and harmonization of mixture risk assessment [19,52,87,89]. This is already evident in practice, as shown for instance by EFSA cumulative assessments of thyroid- and nervous-system-acting pesticides, as well as by cumulative MOE analyses of mycotoxin co-exposures and recent HBM-based applications.

These limitations reinforce the importance not only of methodological refinement, but also of clear communication strategies that transparently convey uncertainty, assumptions and the conditional nature of mixture-based conclusions.

### 6.4. Communication of Mixture Risk and Uncertainty

Decision-useful communication of uncertainty requires formats that are technically sound, yet accessible to managers and the public. Best practice combines qualitative uncertainty matrices (e.g., high/medium/low confidence by domain), quantitative ranges or confidence intervals, and probabilistic outputs when data permit [87,89]. Guidance from EFSA and the National Academies emphasizes the need to distinguish variability (real differences between individuals or scenarios) from uncertainty (lack of knowledge), and to avoid presenting central estimates without explicit context [85,86,89]. Spiegelhalter (2017) [90] highlights the usefulness of visual tools (e.g., probability bands, contribution plots) and carefully chosen analogies to convey the meaning of probabilities, margins of exposure and “safety factors” without oversimplifying. Consequently, reporting binary outcomes (e.g., HI < 1) is insufficient. Effective communication requires visualizing distribution tails and explicit confidence intervals to convey the robustness of safety margins [87,89,90].

Finally, communication should employ clear narratives, contribution plots and intuitive analogies to explain mixture metrics and uncertainty while maintaining scientific accuracy, thereby supporting informed, transparent decision-making on the management of chemical “cocktails” in food and other exposure contexts [87,89,90,109].

### 6.5. Forward-Looking Agenda/Future Priorities

At the same time, current EU research has highlighted several research priorities to improve mixture risk assessment: more efficient and transparent methods to quantify uncertainty (from structured expert judgement through to full probabilistic modelling), empirical validation of key mixture models (dose vs. response addition; interaction bounds) using realistic combinations, and the development of mixture-aware biomarkers and exposure metrics that link external exposure, internal dose and early biological effects [19,50,53,87]. AI and machine-learning approaches may help mine large monitoring and biomonitoring datasets, identify co-exposure patterns, propose dynamic CAGs and accelerate PBPK simulations, but they introduce their own layers of model uncertainty and bias that must be explicitly characterized and validated before regulatory use [19,85,86,87,89].

Thus, over the 2010–2025 period, mixture risk assessment has matured into a tiered, increasingly harmonized practice. Pragmatic anchors like HI and MOET have been operationalized within regulatory frameworks: HI provides a transparent, conservative screen when health-based guidance values are available, whereas MOET enables refined, POD-based aggregation without compounding assessment factors [1,21]. For groups with well-characterized similar modes of action, RPF/TEQ approaches allow potency-scaled summation and facilitate comparison with established reference values [1,2]. MCR adds a diagnostic lens, indicating whether cumulative risk is dominated by one or a few substances or is more evenly distributed, thereby guiding prioritization and the depth of modelling needed [21]. Across major bodies, EFSA, EPA and WHO/FAO now converge on dose addition as the default model for chemicals with similar or overlapping effects, embedded in tiered frameworks that progress from conservative deterministic screening to refined probabilistic assessments, and that require explicit uncertainty analysis at each stage [1,2,86,87].

Data streams have also diversified. Total Diet Studies and Human Biomonitoring now provide complementary perspectives: TDS deliver population-level, “as eaten” exposure estimates for large panels of chemicals, while HBM captures internal doses, integrates all exposure routes and reflects toxicokinetic variability [6,50,53]. When integrated with PBPK modelling, these streams allow source attribution (diet vs. non-diet), cross-validation of exposure models and reconstruction of intakes from biomarker distributions for use in HI or MOET calculations [6,50,53]. Case studies on pesticide CAGs and mycotoxin co-exposures illustrate that cumulative methods can both reassure (showing substantial margins of safety despite multi-residue exposure) and redirect attention (revealing combined concerns not apparent from single-chemical evaluations), if uncertainties are transparently characterized and communicated [67,87,89,124].

Looking ahead, a practice agenda for the next decade rests on five pillars: (i) question-driven problem formulation; (ii) mechanistic grouping; (iii) fit-for-purpose metric selection; (iv) harmonized POD derivation; and (v) integration of TDS, HBM, and PBPK data streams [1,2,6,50,53,87]. On the research side, priorities include defining mixture-sensible thresholds and action levels, achieving realistic LOQs for complex food matrices, validating biomarkers for emerging hazards, and building adaptive, data-driven systems that can update mixture assessments as new monitoring, HBM or toxicological information becomes available [19,87,89]. Together, these elements mark the transition from exploratory mixture science to an operational, uncertainty-aware framework capable of supporting proportionate and transparent public-health decisions in the face of complex dietary chemical mixtures.

## 7. Conclusions and Future Outlook

From 2010 to 2025, mixture risk assessment for food xenobiotics has transitioned from a largely theoretical concern into a structured, operational domain within food-safety regulation. Three pillars underpin this maturation. First, toxicological theory and empirical evidence have consolidated concentration/dose addition as the default predictive model for mixtures of similarly acting chemicals, with large experimental datasets showing that strong synergy or antagonism at dietary exposure levels is relatively rare, especially for pesticide mixtures, even though it cannot be excluded in specific combinations or endpoints [5,12,15,16,17,18,101]. On this basis, a pragmatic portfolio of component-based metrics—HI, RPF/TEQ, MCR and MOET—now allows cumulative risk to be quantified under explicit additivity assumptions, with HI and MCR serving as conservative screening tools and RPF/TEQ–MOET combinations enabling more refined, POD-based assessments when robust mechanistic and dose–response data are available [20,21,32,73,74,75,77,78,80,81].

Second, regulatory frameworks have converged on a tiered, mechanism-informed architecture for cumulative assessment. EFSA’s work on Cumulative Assessment Groups (CAGs) for nervous system, thyroid and craniofacial endpoints, and its 2019 guidance, have embedded CRA in routine EU risk assessment for pesticides, supported by probabilistic exposure platforms such as MCRA and harmonized toxicological databases like OpenFoodTox [1,22,29,30,31,54,55,79,83,84,93]. In parallel, the U.S. EPA has implemented common-mechanism groups and RPF-based cumulative assessments for organophosphate and other pesticides, representing one of the most advanced quantitative applications, albeit focused on domains with strong mechanistic evidence and exposure data [24,25,26,32,80,81]. WHO/FAO frameworks provide globally adaptable principles and stepwise methods that allow countries with heterogeneous capacities to adopt dose-addition-based CRA at proportional levels of complexity [7,23,91,92]. Collectively, these trajectories illustrate a clear shift away from single-chemical paradigms towards more realistic representations of cumulative exposure to food-borne xenobiotics.

Third, exposure science has diversified, with Total Diet Studies (TDS) and Human Biomonitoring (HBM) now forming complementary pillars of mixture assessment. Harmonized TDS designs, refined through WHO/FAO and EFSA guidance and European projects such as TDS-Exposure, provide realistic, “as eaten” estimates of chronic dietary exposure to broad panels of chemicals while maintaining analytical feasibility through composite sampling [34,36,37,38,39,40,41,42,43]. In turn, HBM—via initiatives such as HBM4EU and WHO Europe—quantifies internal doses of PFAS, phthalates, bisphenols, mycotoxins and other contaminants, supports the derivation of HBM guidance values, and links biomarker concentrations with dietary and non-dietary sources through PBPK modelling and reverse dosimetry [6,8,47,50,51,52,53,54,55,58,59,60,61,62,65,103]. Integrated use of TDS, HBM and PBPK models increasingly allows triangulation of exposure, validation of mixture metrics such as HI and MOET against internal dose indicators, and improved attribution of dietary versus non-dietary contributions.

Despite this progress, significant challenges and uncertainties remain. Parameter uncertainty persists due to incomplete toxicological datasets, heterogeneous POD derivation, co-exposure correlations and toxicokinetic variability; model uncertainty stems from metric selection, additivity assumptions and cross-species or route extrapolations; and scenario uncertainty reflects choices in problem formulation, grouping, population subgroups and exposure windows [20,21,85,86,87,88]. Evidence from sensitivity analyses shows that cumulative risk is often dominated by a small subset of “risk-driver” chemicals, even when hundreds of substances are included, underscoring the need for targeted refinement of exposure and hazard data for these drivers and for explicit communication of residual uncertainty from the long tail of co-exposures [67,87,124]. At the same time, data gaps for vulnerable groups, emerging hazards (e.g., microplastics, novel PFAS, nanomaterials), non-persistent compounds and mixture-sensitive biomarkers constrain the scope of current assessments [49,50,66,117].

Looking ahead, the practice agenda relies on five prerequisites: (i) robust problem formulation; (ii) mechanistic grouping; (iii) harmonized POD derivation; (iv) fit-for-purpose metric selection; and (v) data integration [1,14,19,32,77,80,81,87,93,101]. At the data level, sustained investment in TDS and HBM infrastructures, harmonized reporting (e.g., FoodEx2 coding; MIR for HBM), and cross-linkage with PBPK and exposomic tools will be essential to support mixture-aware surveillance and evaluation of risk-management effectiveness [34,41,42,50,53,123]. Research priorities include defining mixture-sensible benchmarks and action levels, improving quantification of interactions within dose-additive bounds, validating exposure and effect biomarkers for emerging contaminants, and harnessing AI and machine learning for data-driven grouping and co-exposure pattern recognition—subject to rigorous validation and transparent handling of new layers of model uncertainty [12,19,53,87,89].

If these strands are integrated and sustained, mixture risk assessment in food safety can fully move beyond the single-compound paradigm toward transparent, tiered, and uncertainty-aware frameworks capable of managing real-world dietary “cocktails” in a proportionate manner [1,7,23,82].

## Figures and Tables

**Table 1:** Key parameters and metrics used in toxicity evaluation and chemical mixture risk assessment, with their role and interpretation in cumulative frameworks.

Parameter/Metric	Definition/Meaning	Role in Mixture Risk Assessment	Notes on Interpretation/Uncertainty
**Point of Departure (POD) (NOAEL/BMDL)**	Toxicological starting point derived from key experimental or epidemiological evidence	Basis for deriving HBGVs and for POD-based aggregation approaches such as MOET	Influenced by study quality, endpoint selection and modelling approach
**Health-Based Guidance Values (ADI/TDI/HBGV)**	Chronic reference values representing levels considered tolerable over lifetime exposure	Used to normalise exposure in Hazard Quotient and Hazard Index calculations	Derived using default assessment factors; residual uncertainty depends on the completeness of the evidence base
**Assessment/Uncertainty Factors**	Default safety factors accounting for interspecies variability, human variability and data gaps	Embedded in reference values and influence apparent risk margins	May obscure underlying uncertainty if applied mechanically; relevant when invoking precaution
**Grouping criteria and justification (CAGs/CKGs)**	Scientific rationale defining which substances are assessed together (shared toxicological mode of action or kinetic behaviour)	Determines mixture composition and applicability of dose-addition models	Misclassification can bias risk estimates; requires transparent documentation and mechanistic support
**Hazard Index (HI)**	Sum of individual hazard quotients (exposure/HBGV)	Provides a conservative, screening-level indicator of cumulative risk	Often misinterpreted as binary “safe/unsafe”; interpretation must consider uncertainty and susceptible groups
**MOET (Margin of Exposure Total)**	Aggregated margin of exposure derived from POD-based estimates	Enables refined cumulative assessment without compounding assessment factors	Dependent on robustness of PODs and study evidence
**RPF/TEQ Approaches**	Potency scaling relative to an index compound	Supports dose-additive aggregation for groups with similar mode of action	Requires strong toxicological justification
**MCR (Maximum Cumulative Ratio)**	Ratio indicating whether cumulative risk is driven by few or multiple contributors	Helps identify “risk drivers” and prioritise monitoring and management	Describes mixture structure rather than absolute risk
**Exposure concentrations in food**	Measured or modelled occurrence levels in relevant food commodities	Core input for cumulative dietary exposure estimation	Affected by analytical uncertainty, detection limits and data representativeness
**Consumption distributions (including high percentiles)**	Population-level food intake variability	Determines distribution of exposure and risk across the population	High-percentile estimates are sensitive to data quality and survey design
**Internal biomarker concentrations (HBM)**	Measured biomarkers reflecting internal exposure levels	Support estimation of internal dose and validation of exposure models	Influenced by kinetics, sampling time and population variability
**PBPK modelling inputs**	Toxicokinetic parameters linking external intake to internal dose	Support interpretation of HBM and reconstruction of intake for CRA metrics	Parameter and model uncertainty affect confidence in back-calculations
**Co-exposure correlations**	Dependence between exposures to different chemicals	Influences cumulative exposure estimates and realism of models	Often neglected; contributes to structural uncertainty
**Assessment scenario parameters (population subgroup, time window, regulatory framing)**	Definition of target population, exposure duration and regulatory question guiding assessment	Shapes which exposures are included and how results inform decision-making	Scenario choices introduce “scenario uncertainty”; selection of vulnerable groups is critical

**Table 2:** Comparative analysis of regulatory frameworks for cumulative risk assessment.

DIMENSION	EFSA	U.S. EPA	FAO&WHO
**Scope**	Pesticides, contaminants, risk-benefit	Pesticides (primary); mechanism groups	Multi-hazard; scalable globally
**Primary Metrics**	HI, CAGs, MOET, MCR	RPF, CMG, MOE, BMD-POD	HI, RPF, MOET, GV
**Grouping**	Effect-based CAGs (thyroid, neuro, dev)	MOA-based (OP, triazoles)	MOA/effect-based; flexible
**Legal Status**	Mandatory (EU Food Law 2006/88/EC)	Binding (FQPA mandate)	Advisory (Codex framework)
**Data Platform**	MCRA, OpenFoodTox, EFSA Warehouse	ToxCast, ExpoCast, CompTox	FAO/WHO tools, proportionate methods
**Decision Rule**	HI > 1 then signal; MOET ≥ 100–10,000	MOE/RPF < 100–10,000 (potency-adjusted)	HI > 1 then refine; regional flexibility
**Strengths**	Probabilistic rigor; legally enforced; TDS-integrated	Pesticide expertise; RPF well-established; regular updates	Globally applicable; capacity-scalable; multi-hazard inclusive
**Challenges**	Data-intensive; CAG construction subjective	Narrow scope (mainly pesticides); limited non-pesticide framework	Non-binding; variable uptake; limited resources in developing countries
**Future**	PBPK modelling; PFAS expansion; HBM integration; RACEMiC harmonization	HTS/computational toxicology; RPF expansion (PFAS, flame retardants); refined exposure models	EFSA/EPA alignment; HBM integration; regional capacity building; data infrastructure

**Table 3:** Applicability and prioritisation of key methodologies in cumulative mixture risk assessment.

Methodology/Metric	Primary Purpose	Applicability Contexts	Key Limitations/Constraints
Hazard Index (HI)	Conservative screening of cumulative risk using HBGVs	Early tier assessments; large groups; regulatory signalling thresholds	May appear binary; masks uncertainty; assumes adequate HBGVs
MOET (Margin of Exposure Total)	Refined cumulative risk estimation using PODs	When more robust toxicological data are available; moving beyond screening	Depends on POD quality; requires harmonised derivation
RPF/TEQ Approaches	Potency-based aggregation for chemicals with similar MoA	Well-characterised chemical groups; strong mechanistic justification	Not suitable when MoA similarity is uncertain
MCR	Diagnosing mixture structure and prioritising drivers	Identifying whether risk is dominated by few or multiple compounds; guiding management	Does not provide absolute risk estimate
Probabilistic models/sensitivity analysis	Quantifying variability and uncertainty	When data availability supports refined assessment and policy relevance	Data-demanding
HBM + PBPK integration	Linking external exposure to internal dose and validating models	When biomonitoring data exist; policy-focused contexts	Requires biomarkers + kinetic knowledge

## Data Availability

No new data were created or analyzed in this study. Data sharing is not applicable to this article.
